# Transcriptome analysis of the pectoral muscles of local chickens and commercial broilers using Ribo-Zero ribonucleic acid sequencing

**DOI:** 10.1371/journal.pone.0184115

**Published:** 2017-09-01

**Authors:** Yanhua Zhang, Donghua Li, Ruili Han, Yanbin Wang, Guoxi Li, Xiaojun Liu, Yadong Tian, Xiangtao Kang, Zhuanjian Li

**Affiliations:** 1 College of Animal Science and Veterinary Medicine, Henan Agricultural University, Zhengzhou, China; 2 Henan Innovative Engineering Research Center of Poultry Germplasm Resource, Zhengzhou, China; Kunming University of Science and Technology, CHINA

## Abstract

**Background:**

The molecular mechanisms underlying meat quality and muscle growth are not clear. The meat quality and growth rates of local chickens and commercial broilers are very different. The Ribo-Zero RNA-Seq technology is an effective means of analyzing transcript groups to clarify molecular mechanisms. The aim of this study was to provide a reference for studies of the differences in the meat quality and growth of different breeds of chickens.

**Results:**

Ribo-Zero RNA-Seq technology was used to analyze the pectoral muscle transcriptomes of Gushi chickens and AA broilers. Compared with AA broilers, 1649 genes with annotated information were significantly differentially expressed (736 upregulated and 913 downregulated) in Gushi chickens with Q≤0.05 (Q is the P-value corrected by multiple assumptions test) at a fold change ≥2 or ≤0.5. In addition, 2540 novel significantly differentially expressed (SDE) genes (1405 upregulated and 1135 downregulated) were discovered. The results showed that the main signal transduction pathways that differed between Gushi chickens and AA broilers were related to amino acid metabolism. Amino acids are important for protein synthesis, and they regulate key metabolic pathways to improve the growth, development and reproduction of organisms.

**Conclusion:**

This study showed that differentially expressed genes in the pectoral tissues of Gushi chickens and AA broilers were related to fat metabolism, which affects meat. Additionally, a large number of novel genes were found that may be involved in fat metabolism and thus may affect the formation of meat, which requires further study. The results of this study provide a reference for further studies of the molecular mechanisms of meat formation.

## Introduction

The transcriptome is a necessary link connecting genomic/genetic information with the biological functions of the proteome. RNA sequencing (RNA-Seq) is typically used for transcriptome profiling and for analyzing transcript isomers, variable splicing and genomic structural variations. Generally, the preparation of RNA-Seq libraries is based on the specificity of oligo(dT) primers binding to poly(A)+ transcripts to remove ribosomal RNA (rRNA) from total RNA; this method relies on the integrity of the total RNA and the efficient removal of ribosomal RNA[1]. PolyA-Seq has been used to study the transcriptomes of different tissues or cells from animals, including cattle, sheep, chickens, and pigs[2–5]. However, based on the principles of PolyA-Seq, it is rarely used to study non-poly(A) transcripts or partially degraded mRNAs, although poly(A)-RNAs, both protein-coding and noncoding, are functionally important[6, 7]. The superiority of the Ribo-Zero method is its ability to capture poly+ and poly- transcripts rapidly from intact or partially degraded RNA samples, including coding RNA and various forms of noncoding RNA[8, 9]. The development of sequencing technology has enabled richer transcript information to be obtained.

The high demand for meat and egg production has led to breeding efforts in the past few decades focused on increasing the growth of the chest and leg muscles while ignoring the improvement of meat quality. Meat quality is associated with various factors, including breed, sex, age, and organizational differences, of which breed is an important factor. Influenced by traditional food culture, consumers favor the unique flavor characteristics of local chicken[10]. Meat quality is a comprehensive concept that includes flavor, tenderness, color and other factors. The intramuscular fat (IMF) content of chicken meat is an important element of consumer preference due to its positive correlation with meat quality, including tenderness and juiciness. IMF involves the muscle fibers distributed in the muscle tissue, especially in chickens, where IMF is mainly distributed in the epimysium, perimysium, and endomysium. IMF improves the tenderness of the meat by making the muscles soft and juicy through a reduction in muscle shear; thus, the IMF content and muscle tenderness are significantly positively correlated[11]. Adipocyte differentiation and lipid metabolism play an important role in the deposition process of IMF, which involves a series of changes in the regulation of different transcription factors and the regulation of genes that control lipid metabolism[12]. Transcription factor activity is regulated by a variety of intracellular and extracellular factors and signaling pathways, which play decisive roles in the differentiation of precursor adipocytes into mature adipocytes[13]. The generation of poultry fat is different from the generation of mammalian fat because chicken fatty acids are mainly synthesized in the liver. Lipoproteins hydrolyze and release fatty acids that have been transported into adipose tissue, then synthesized and deposited in fat cells[14]. Research into the mechanisms of molecular regulation of fat generation is key to understanding the molecular mechanisms of meat quality formation.

Improving meat quality while considering yield is the focus of current breeding research. In local chickens, the IMF content is rich and evenly distributed, which produces delicious meat. The objective of this study was to investigate differentially expressed genes (DEGs) closely related to both the meat quality (IMF content) and the growth of chickens using strand-specific Ribo-Zero RNA-Seq to compare Gushi chickens (local chickens) and AA broilers (large-scale commercial broilers).

## Materials and methods

### Experimental animals and tissue collection

The two chicken breeds used in this study were both obtained from the Animal Center of Henan Agricultural University. Three 6-week-old birds were randomly selected from each breed (Gushi and AA) and killed by stunning followed by exsanguination. Pectoral tissues were removed quickly, rinsed with DEPC water, snap-frozen in liquid nitrogen, and stored at -80°C until use.

### Ethics statement

All animal experiments were performed in accordance with the protocol approved by the Institutional Animal Care and Use Committee (IACUC) of Henan Agricultural University and were approved by the Animal Care Committee of the College of Animal Science and Veterinary Medicine, Henan Agricultural University, China (Permit Number: 11–0085). All efforts were made to minimize animal suffering.

### Ribo-Zero RNA sequencing

#### Library construction and sequencing

Total RNA was isolated from the pectoral tissues of chickens using an RNeasy Micro Kit (QIAGEN, Hilden, Germany) and treated with DNase (QIAGEN) to remove DNA. The purity of the total RNA was assessed with a NanoDrop ND-1000 spectrophotometer (NanoDrop, Wilmington, DE, USA). The integrity of the total RNA was estimated using an RNA Nano 6000 Assay Kit with the Agilent 2100 Bioanalyzer (Agilent Technologies, Santa Clara, CA, USA). The degradation and contamination of total RNA was detected using 1% agarose gels. Three pectoral muscle RNA samples from each breed were pooled to obtain an “average” transcriptome. The Illumina TruSeq RNA Sample Prep Kit (Illumina, Inc., San Diego, CA, USA) was used to prepare the Ribo-Zero RNA-Seq libraries according to the manufacturer’s protocol. Then, rRNA was removed from the total RNA with the Ribo-Zero rRNA Removal Kit (Epicentre, Madison, WI, USA). The total RNA without rRNA was purified, fragmented, and primed for cDNA synthesis. A Qubit® 2.0 Fluorometer (Invitrogen) and an Agilent 2100 Bioanalyzer (Agilent Technologies) were used to confirm the quality and concentration of the 2 libraries. Adapter-ligated cDNA fragment libraries were analyzed using paired-end 2× 100 nt Illumina HiSeq 2500 controlled by data collection software. The image data were outputted and transformed into raw reads and stored in FASTQ format after sequencing. Raw read sequencing data may contain failed reads and sequencing primers, which lower the overall quality. These failed reads would likely have some effect on the quality of the analysis; therefore, they were filtered using the FASTX Toolkit (version: 0.0.13)[15] to obtain clean reads for use in the data analysis. Low-quality bases with Q-values <20 (i.e., with a base error rate less than 0.01, where Q = -10logerror_ratio) were removed from the 3' end. Linker sequences, reads shorter than 20 nt, and ribosomal RNA reads were removed. The resulting clean reads were used in the downstream analyses.

#### Mapping, assembly, and transcript abundance estimation

The spliced mapping algorithm of TopHat (version: 2.1.0)[16] was used to perform genome mapping on the preprocessed reads. The clean reads were mapped to the chicken genome assembly (*Gallus gallus* 5.0) from Ensemble. TopHat is particularly suitable for analyzing eukaryotic (intronic and intermolecular) transcriptome sequencing data because it allows comparisons of reads without full-length matches and consummate catalogs of alternative splicing events[17]. Based on the TopHat comparison results, the data for each library were spliced and assembled using Cufflinks (version 2.1.1). The Cuffcompare program in the Cufflinks package compares Cufflinks assemblies to reference annotation files and helps sort out novel genes from the reference notes. The gene expression data were standardized by being converted into FPKM (fragments per kilobase of exon model per million mapped reads) to make the expression levels comparable between different genes and samples[18]. After the TopHat alignment, we used HTSeq[19] to count the number of fragments for each gene and then normalized them with TMM (trimmed mean of M values)[20]; finally, we used a Perl script to calculate the FPKM values using the following formula:

$$FPKM=\frac{total\;exon\;fragments}{mapped\;reads\;(millions)\times exon\;length\;(kb)}$$

#### Identification and functional annotation of differentially expressed genes

We analyzed the known and novel genes using edgeR[21] and determined the threshold of the P-value by the false discovery rate (FDR) in multiple tests and analyses[22, 23]. The Q-value was used to denote the regulated P-value. We identified DEGs as genes with a Q-value ≤0.05 and a fold change (FC) ≥2 or ≤0.5 (log_2_FC≥1 or ≤-1).

DAVID software[24] was used to analyze the functional enrichment of DEGs and to identify enriched Gene Ontology (GO: http://www.geneontology. org) and Kyoto Encyclopedia of Genes and Genomes (KEGG: http://www.kegg.jp/) pathways. By comparing the DEGs with the genomic background in the GO database, all the significantly enriched GO terms of the DEGs that corresponded to biological functions were obtained. The KEGG pathway enrichment analysis of DEGs was performed to identify enriched pathways and clarify the breed differences in cellular pathways. The following formula was used to filter for enrichment of GO terms:

P=1−∑i=0m−1(Mi)(N−Mn−i)(Nn)

where N indicates the total number of genes in the GO terms, n indicates the number of DEGs in N, M indicates the number of genes in a specific GO term, and m indicates the number of SDE genes annotated with the same specific GO term. GO terms and KEGG pathways with P≤0.05 were identified as significantly enriched among the SDE genes. The KEGG analysis was the same as the GO term analysis.

#### Quantitative real-time PCR (qRT-PCR) validation of RNA sequencing data

To confirm the reproducibility and accuracy of the RNA-Seq gene expression data, 15 genes (including 9 upregulated and 6 downregulated genes) were randomly selected for quantitative real-time PCR in the two breeds. Using RNAiso Plus (TaKaRa, Dalian, China), total RNA was extracted from the pectoral muscles of Gushi chickens and AA broilers and used in library construction; 3 biological replicates were performed. Then, first-strand cDNA was synthesized using the PrimeScript™ RT Reagent Kit with gDNA Eraser (TaKaRa, Dalian, China).

The qRT-PCR was performed in a 25-μL reaction volume containing 2 μL (approximately 100 ng) of cDNA, 12.5 μL of 2× SYBR® Premix Ex Taq^TM^ II (Tli RNaseH Plus) (TaKaRa), 0.5 μL each of the forward and reverse primers (10 μM), and 7 μL of deionized water on a LightCycler® 96 Real-Time PCR system (Roche Applied Science). The relative gene expression levels were calculated with the comparative CT method (also referred to as the 2^-△△CT^ method)[25] using GAPDH as the reference gene. The qRT-PCR amplification procedure was as follows: 95°C for 3 min; 40 cycles of 95°C for 12 s, 61°C for 40 s, and 72°C for 30 s; and extension at 72°C for 10 min. The primers are shown in the S1 Table. Statistical analyses of the data were performed using SPSS (version 21.0; SPSS Inc., Chicago, IL, USA). One-way and repeated-measures analyses of variance were performed, followed by Dunnett’s test. A significance level of P≤0.05 was set. The data are presented as the means ± SEM.

## Results

### Whole-transcriptome deep sequencing of chicken pectoral muscle using Ribo-Zero RNA sequencing

**Fig 1:**
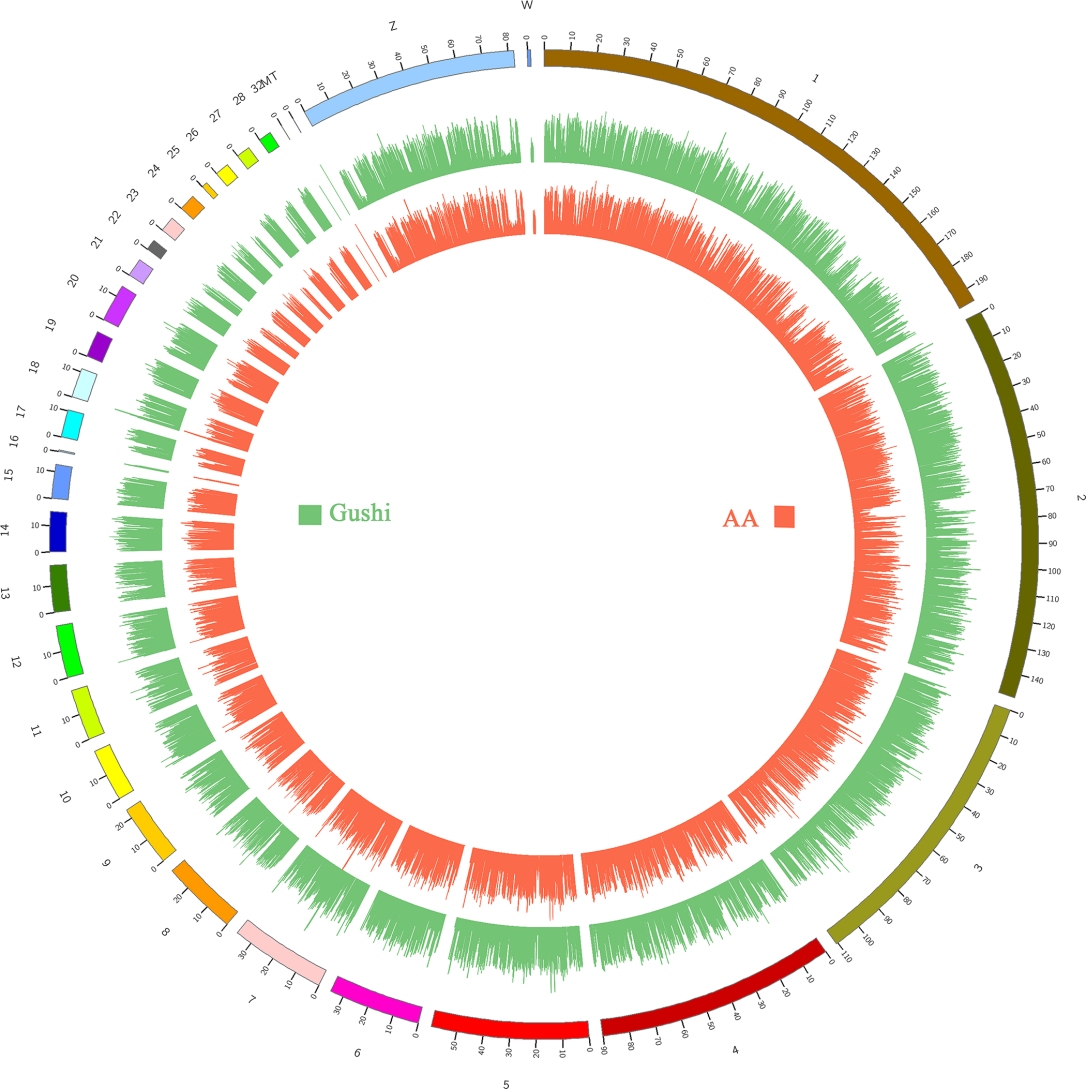
Genome coverage map. The outermost circle is the genome, the first inner circle shows the chromosome coverage of the AA chicken (AA), and the second shows that of the Gushi chicken (Gushi). The number on the outer circle represents the chromosome number, the number on the inner circle represents the position on the chromosome, and the unit is Mb.

**Fig 2:**
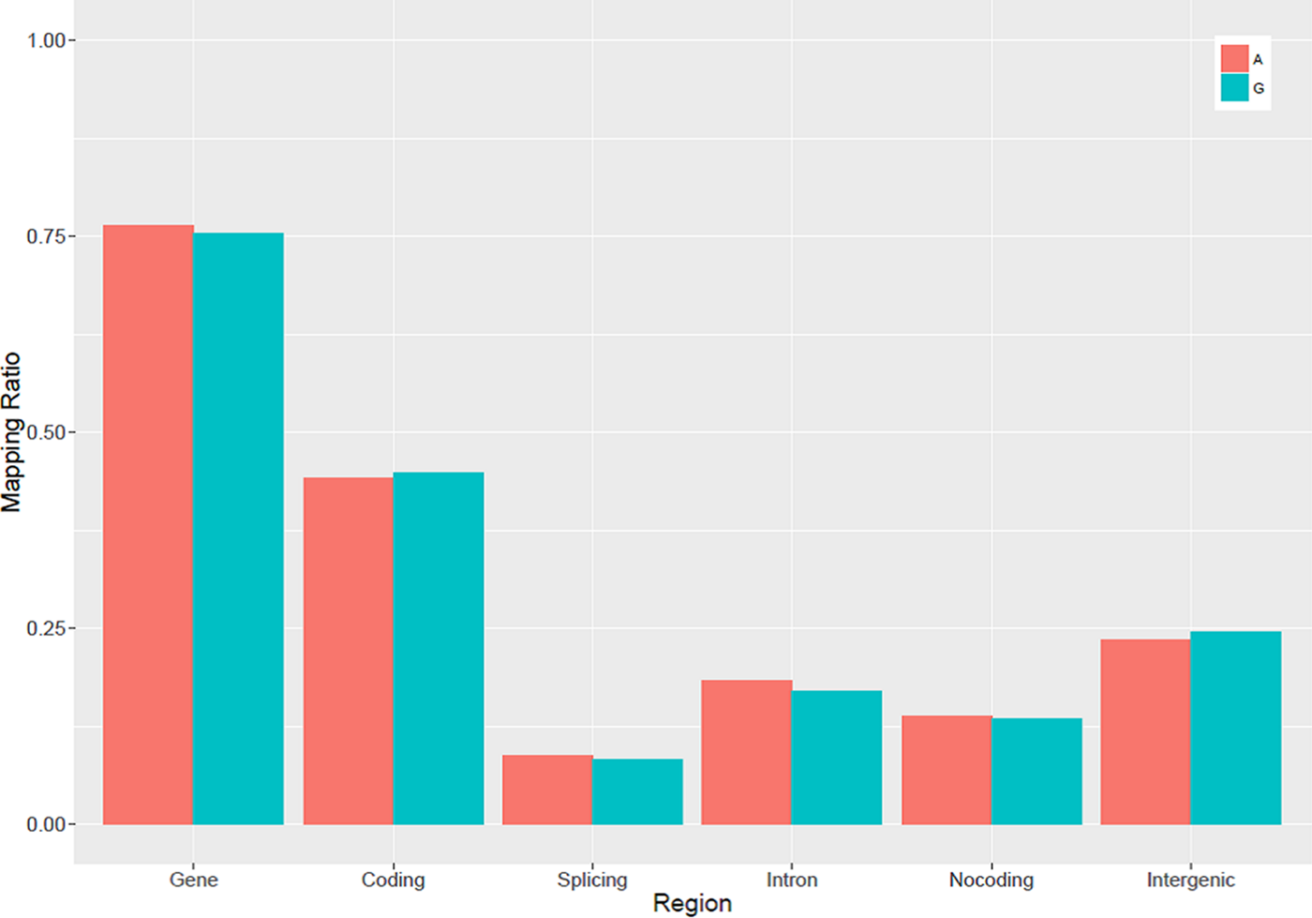
Distribution of the mapped reads on different regions of the chicken reference genome. Noncoding regions include all the 5′UTR, 3′UTR and other noncoding RNA regions. UTR = untranslated region.

The Ribo-Zero RNA-Seq libraries of the two breeds were sequenced on the Illumina HiSeq 2500 platform, generating 194 million raw paired-end reads. After the low-quality reads were filtered, 113,689,278 and 70,137,370 clean reads were obtained for Gushi chickens and AA broilers, respectively. These 184 million (total) clean reads were subjected to further analysis; 102,625,185 (90.3%) and 64,426,854 (91.9%) reads for the Gushi chickens and AA broilers, respectively, were mapped to the chicken reference genome. The average mapping frequency was 91.1% alignment to the chicken reference genome. The frequency for the Gushi chickens was lower than that for the AA broilers, demonstrating that the local chicken genome is specific and contains unique genetic information (Table 1). On average, 86.4% of the reads were uniquely mapped to the galGal5 assembly of the chicken genome, and the mapping frequencies were 87.3% and 85.5% for the Gushi and AA groups, respectively. Reads were evenly mapped to each chromosome, there was no significant difference between the samples, and the depth was consistent (Fig 1). The mapping of sequencing data to various regions of the genome (Fig 2) revealed that most of reads were mapped to gene and coding regions. The same result was obtained in a previous study [26].

**Table 1:** Characteristics of the reads from pectoral libraries obtained from 2 breeds of chicken.

Samples ID	Raw reads	Clean reads	Mapped reads	Mapped Pair Reads	Mapped Unique Reads^1^	Mapped Multi reads	Mapping ratio^2^	Uniquely mapping ratio^3^
A^4^	70,151,126	70,137,370	64,426,854	60,942,128	61,217,966	3,208,888	91.9%	87.3%
G^4^	113,719,518	113,689,278	102,625,185	96,147,504	97,248,821	5,376,364	90.3%	85.5%

^1^Mapped Unique reads = reads that matched only one position in the genome.

^2^Mapping ratio = mapped reads/clean reads.

^3^Unique mapping ratio = mapped unique reads/clean reads.

^4^G: Gushi chicken, A: AA chicken

### Global genes and alternative splicing transcripts

**Fig 3:**
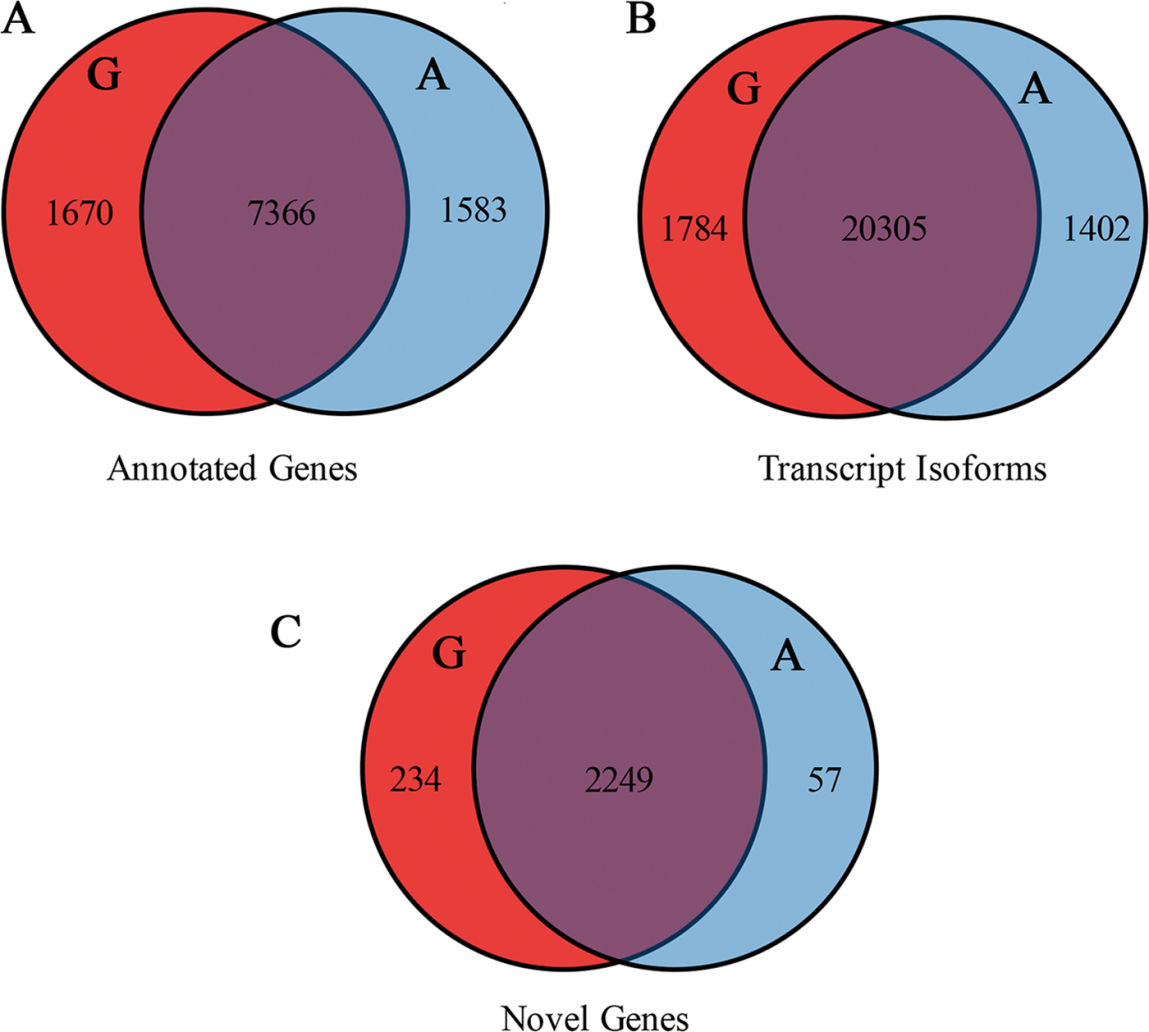
Venn diagram of global annotated genes, transcript isoforms and novel genes expressed in Gushi chickens and AA chickens by Ribo-Zero RNA sequencing. Venn diagrams show the distribution of annotated genes (A), transcript isoforms (B), and novel genes (C) detected in Gushi chickens and AA broilers. Red indicates Gushi chickens, and blue indicates AA chickens.

In the two groups of data, we detected 10,574 genes with annotated information, of which 9036 and 8904 genes were detected in Gushi chickens and AA broilers, respectively. To clarify the regulation of the genes, we examined the transcriptome data and found that 1670 and 1538 genes were expressed in a variety-specific form in Gushi chickens and AA broilers, respectively (Fig 3A). A transcript represents a mature RNA molecule, and "gene" refers to one or more transcript subtypes of a common exon in a mature cut form. When the transcription factor expression level changes, genes with different subtypes can encode the same protein, which helps maintain the gene expression at a certain level[27]. In addition, different subtypes of the same gene can also encode different proteins, which may have specific functions in different breeds. PolyA-Seq has not been used to measure global transcript isoform expression in previous chicken transcriptome analyses[28]. In this study, 23,491 transcript subtypes were detected in the two sequencing libraries, of which 22,089 were detected in Gushi chickens and slightly fewer (21,707) were found in AA broilers. Among all the transcripts, 1784 were specifically expressed in Gushi chickens and 1402 in AA broilers (Fig 3B).

**Fig 4:**
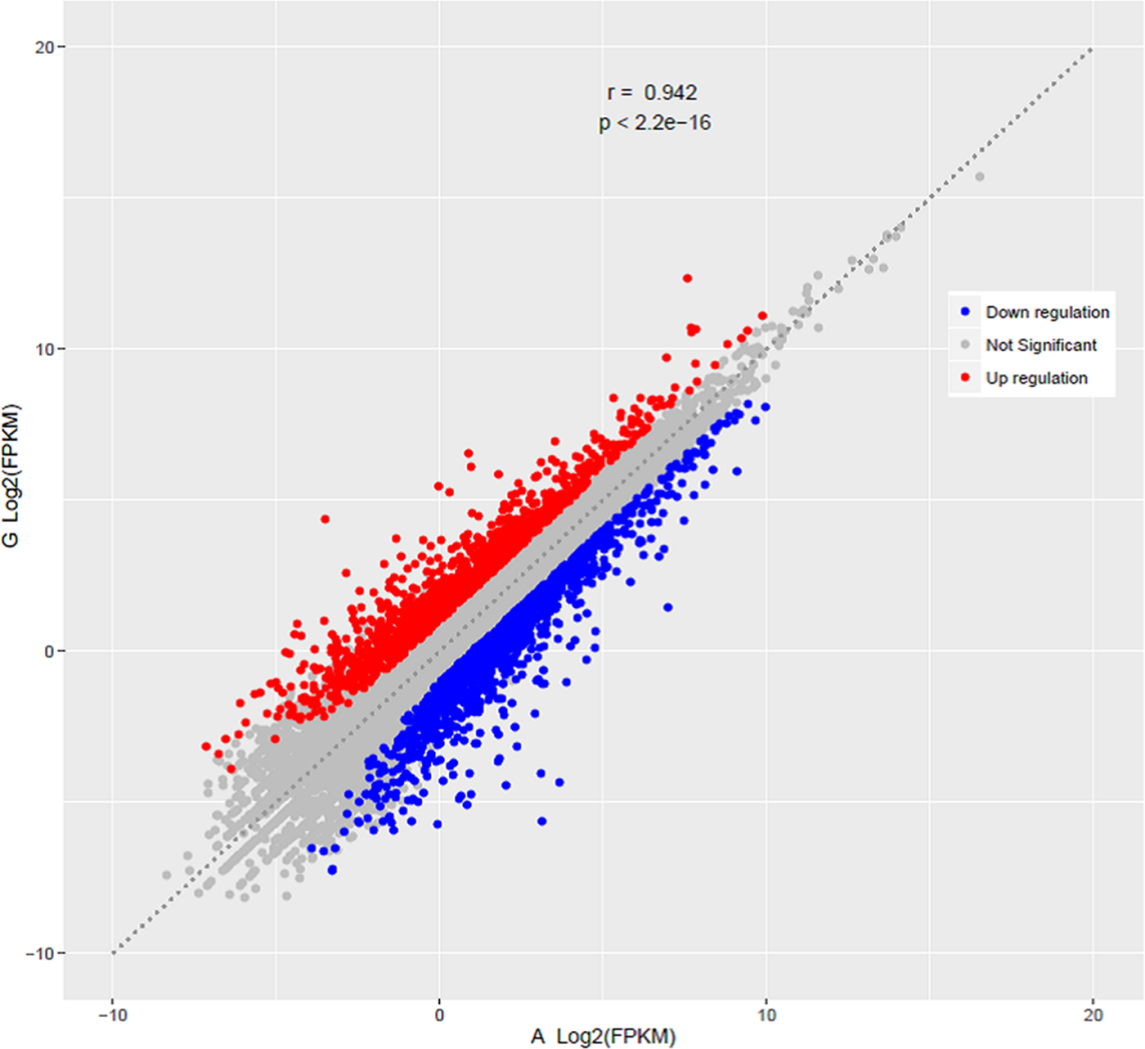
Comparison of differentially expressed genes between the two breeds. Scatter plot showing the correlation of gene abundance. Red points represent genes upregulated by at least two fold at FDR ≤ 0.05, blue points represent genes downregulated at the same thresholds, and grey dots indicate transcripts that did not change significantly.

We analyzed the transcriptome data of the two breeds in more detail and found that the correlation between the two groups of gene expression data was very high, with a correlation coefficient R^2^ = 0.942 (Fig 4). This result indicated that the modeling and experimentation was relatively reasonable. In addition, the majority of the 10 most highly expressed genes was associated with muscle development (S2 Table), and most of the genes were more highly expressed in AA broilers than in Gushi chickens. AA broilers grow more rapidly than Gushi chickens; thus, the high levels of expression of these genes may be necessary to achieve the rapid growth of AA broilers. Therefore, the expression levels of genes related to muscle development, basic biochemical reactions and metabolism in the chest muscles of AA broilers were higher than those of Gushi chickens.

### Identification of differentially expressed genes

The detection and analysis of DEGs between breeds helps elucidate the regulation of genes. We found 2625 significant DEGs in this study, which included 1342 downregulated and 1283 upregulated genes in Gushi chickens compared with AA broilers. Among the significant DEGs, 1649 had annotated information (S3 Table), which included 913 downregulated and 736 upregulated genes in Gushi chickens compared with AA broilers. The S4 Table lists the top 20 significantly up- and downregulated genes between the samples according to the log_2_FC. Among the significantly downregulated genes, lipoprotein lipase (*LPL*), fatty acid binding protein 5 (*FABP5*), and peroxisome proliferator activated receptor gamma (*PPARG*) have been reported to be associated with lipid metabolism[29–31], and myosin light chain 10 (*MYL10*), myosin heavy chain 7B (*MYH7B*), myosin light chain 3 (*MYL3*), and cysteine- and glycine-rich protein 3 (*CSRP3*) are involved in muscle development and associated with growth rates [32, 33]. Significantly upregulated genes included fibroblast growth factor 4 (*FGF4*) associated with muscle development[32] and cholesteryl ester transfer protein (*CETP*) associated with lipid metabolism and deposition[34].

### Functional analysis of differentially expressed genes

We analyzed the functional distribution of the DEGs in the pectoral muscle of local chickens compared with commercial broilers using GO enrichment and KEGG pathway analyses to understand the regulatory network of meat quality formation.

**Fig 5:**
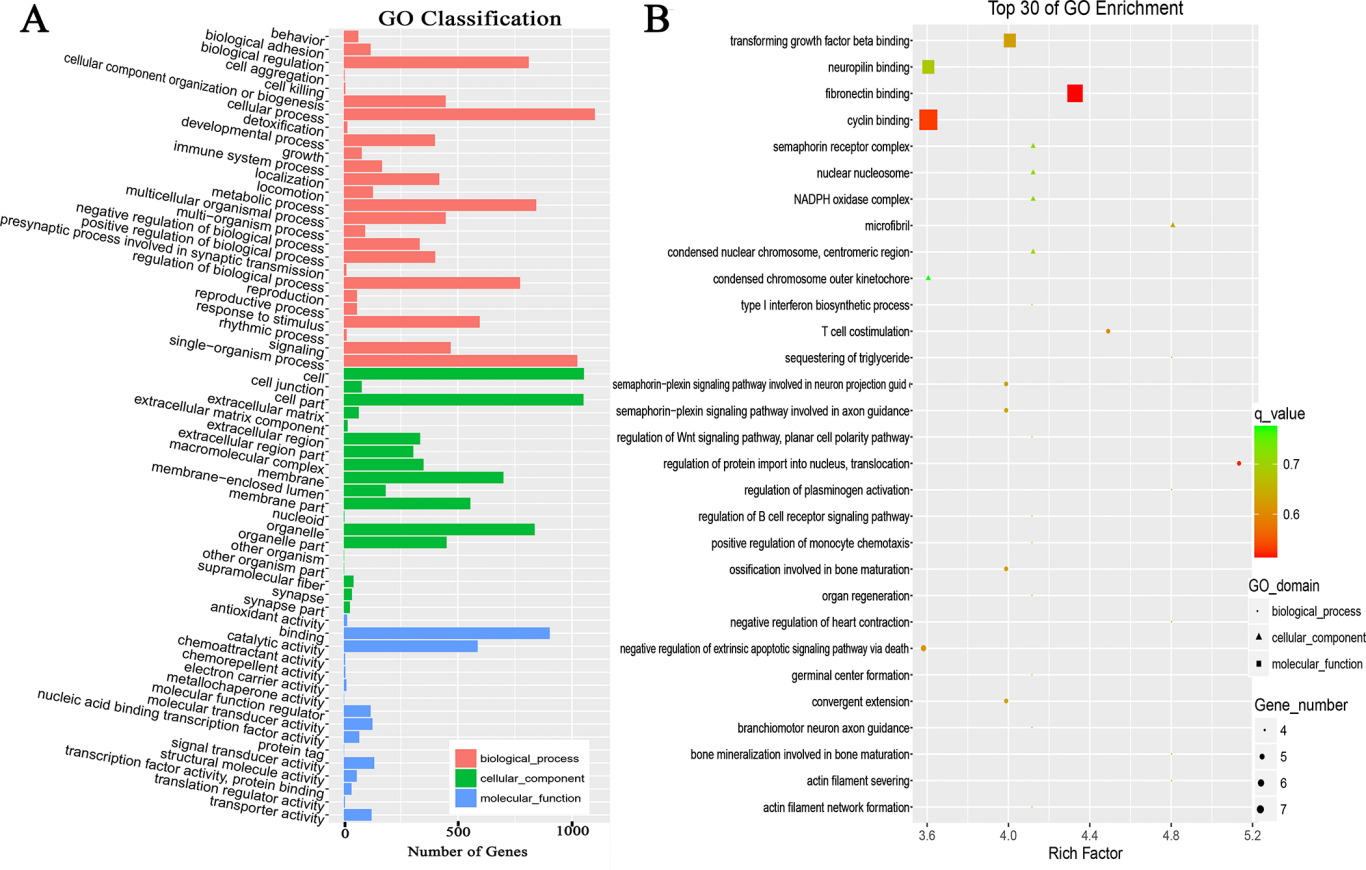
GO analyses of differentially expressed genes in Gushi chickens and AA chickens. A shows the GO function classification (level 2), and B shows the 30 most significantly enriched GO terms.

GO is a type of biological ontology language divided into three parts: biological process, cellular component and molecular function (Fig 5A). Unlike the functional annotation of a single gene, a gene functional enrichment analysis is based on GO entries, and the results can directly reveal the overall functional characteristics of an entire list of genes. The 1408 DEGs of Gushi chickens/AA broilers were enriched for 382 GO terms (S5 Table). The 30 most significantly enriched GO terms are shown in Fig 5B. (For details see S5 Table). The most significantly enriched GO term was the positive regulation of immune system processes. This result is consistent with the different immunity and anti-stress abilities of local chickens and commercial broilers[35].

**Fig 6:**
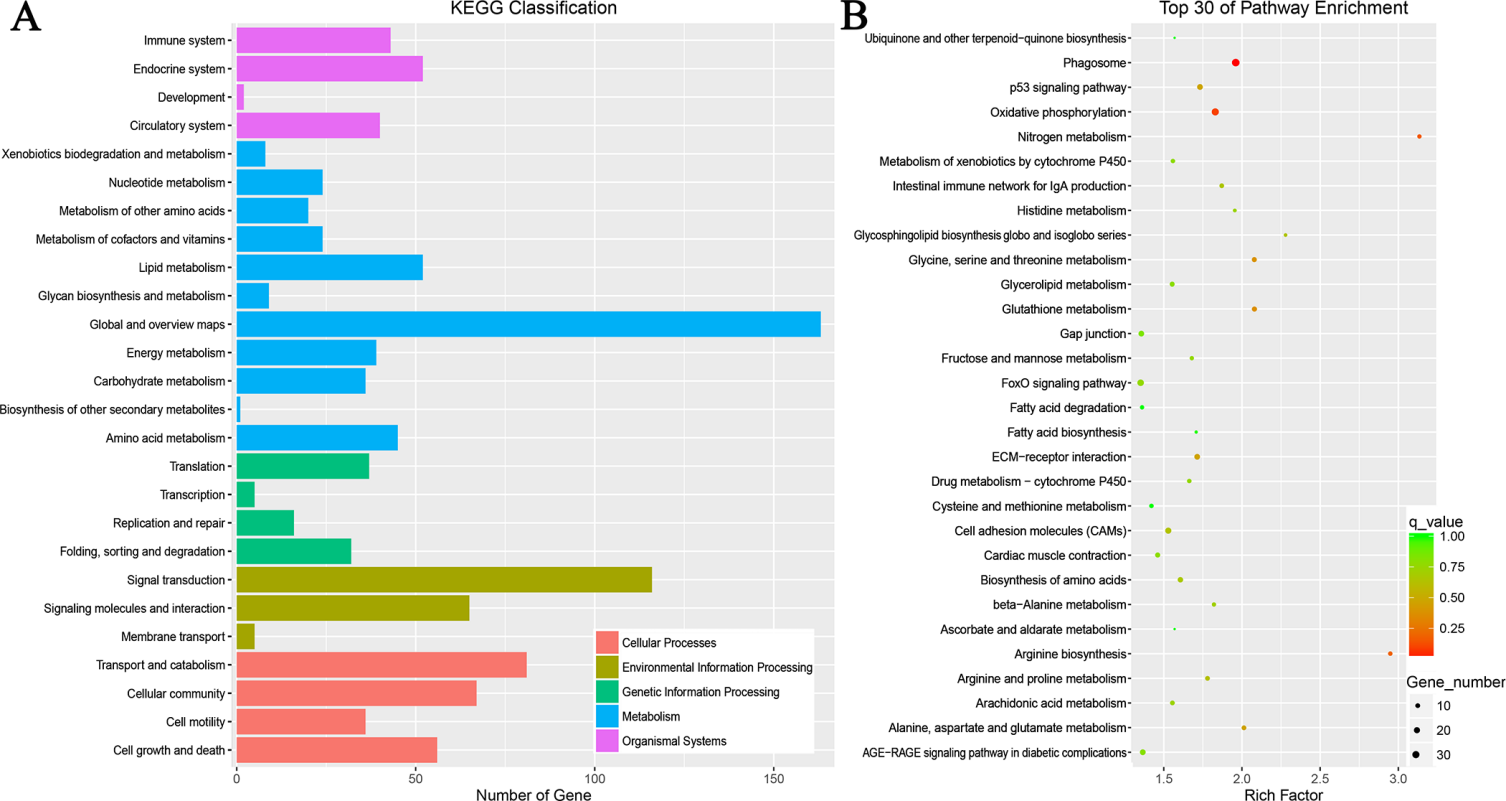
KEGG pathway analyses of differentially expressed genes in Gushi chickens and AA chickens. A shows the KEGG pathway classification, and B shows the 30 most significantly enriched KEGG pathways.

The 614 DEGs of Gushi chickens/AA broilers were annotated into 147 pathways, including five different classifications (Fig 6A), of which 11 were significantly enriched (P≤0.05), and the 30 most enriched are shown in Fig 6B and in S6 Table. The results showed that the signal transduction pathways of Gushi chickens and AA broilers were mainly related to amino acid metabolism, including arginine biosynthesis, nitrogen metabolism, glycine, serine and threonine metabolism, and alanine, aspartate and glutamate metabolism. In addition, enrichment was found in immune-related signaling pathways, including the phagosome, glutathione metabolism, the p53 signaling pathway, ECM-receptor interaction, cell adhesion molecules (CAMs), and the intestinal immune network for IgA production. Amino acids are involved in protein synthesis and regulate key metabolic pathways to improve the health, survival, growth, development, lactation, and reproduction of organisms[36]. Proteins maintain and promote growth and development. Compared with the genes in AA broilers, the Gushi chicken genes in the KEGG-enriched amino acid metabolic pathway were mostly downregulated, which may be related to the rapid metabolism of AA broilers. The enrichment of immune-related signaling pathways is consistent with the different immunity and anti-stress abilities of local chickens and commercial chickens, similar to the GO enrichment results.

### Discovery of novel genes

Using the Cuffcompare software, we detected 12,699 novel genes that contained no annotation information in the Ensemble database. Among these genes, 12,642 were detected in Gushi chickens and 12,465 in AA broilers. A comparison of Gushi chickens with AA broilers revealed 2540 significant DEGs (FC≥2 or ≤0.5, Q-value≤0.05), comprising 1405 downregulated and 1135 upregulated genes, as shown in S7 Table. Among these novel genes, more specific expressed genes were detected in Gushi chickens (234) than in AA chickens (57) (Fig 3C), indicating the need to strengthen the study of the local varieties of Gushi chickens with the characteristics. Most of the novel genes were expressed at low levels or showed stage-specific expression, which may explain why they were barely detected in previous studies. Genes with phase-specific expression, both annotated and non-annotated, exhibited low expression in the present research.

### Analysis of alternative splicing

**Fig 7:**
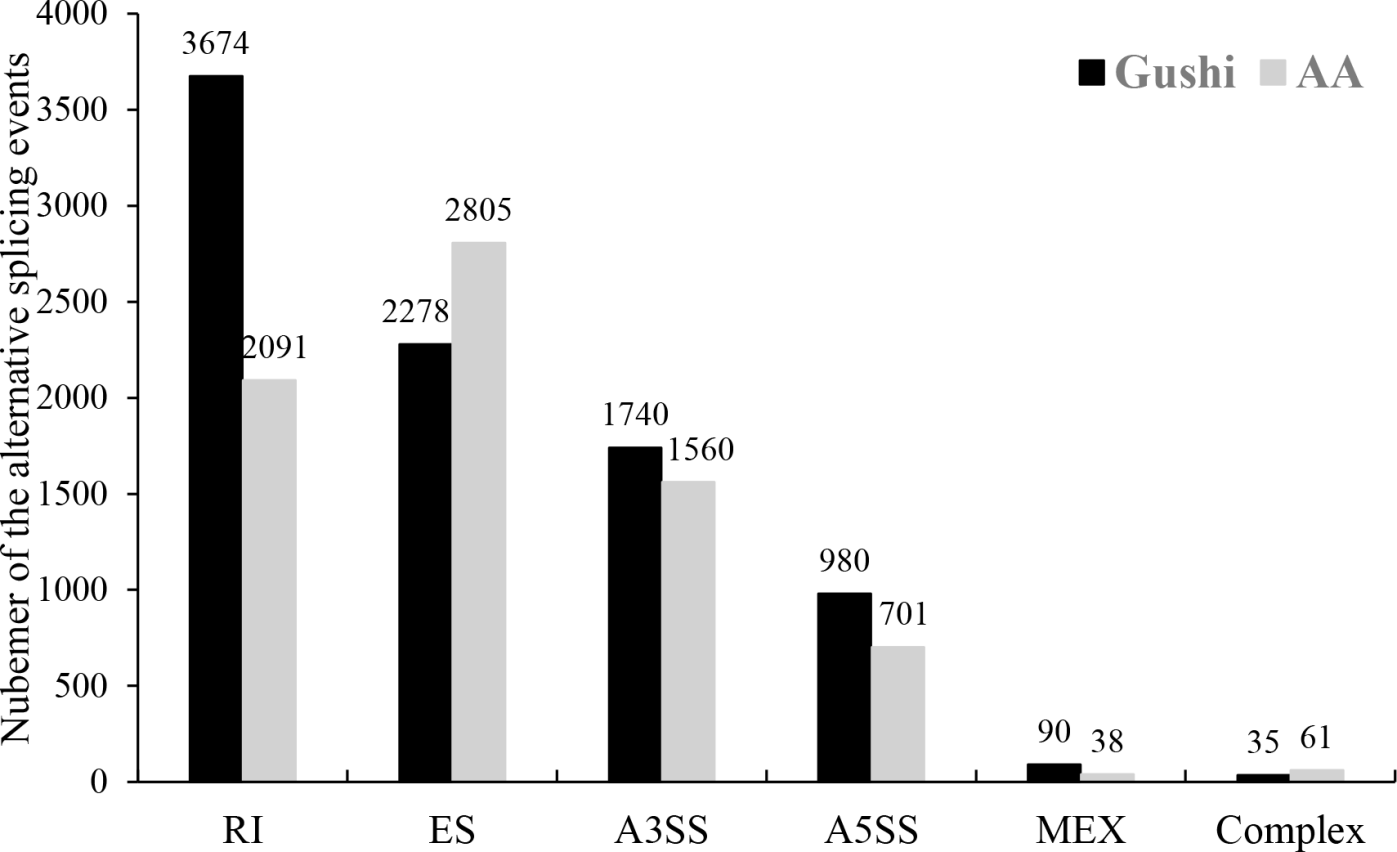
Schematic representation of the alternative splicing events in the samples. The y-value represents the number of observed splicing events, and the x-value shows the major six alternative splicing types in Gushi chickens and AA chickens, respectively. RI = retained introns; ES = exon skipping; A3SS = alternative 3' splicing sites; A5SS = alternative 5' splicing sites; MEX = mutually exclusive exons.

Alternative splicing events in transcripts from the Gushi chickens and AA broilers were analyzed using AStalavista software (version 3.2) [22, 23]. There were 8797 splicing events corresponding to 4008 genes in Gushi chickens and 7256 splicing events corresponding to 3981 genes in AA broilers. To identify the composition of alternative splicing products, we divided all the detected splicing events into six types: alternative 5’ splicing sites, alternative 3’ splicing sites, mutually exclusive exons, exons skipped, retained introns and complex types (Fig 7). Interestingly, the largest number of splicing events in Gushi chickens was retained introns, accounting for 41.76% of the total, while skipped exons were most common in AA broilers, at a proportion of 38.66%. The higher number of splicing events in the Gushi chickens compared with AA broilers demonstrated that the genetic diversity of local chickens (Gushi chickens) is more abundant than commercial chickens (AA chickens).

### SNP and InDel detection

Transcriptional sequences can reflect genetic composition. Therefore, we used SAMtools mpileup[37] [38] to analyze the transcriptome data for single nucleotide polymorphisms (SNPs) and insertions/deletions (InDels). The SNPs were then annotated by snpEff [39]. To achieve high-quality genetic variation, we adopted a series of stringent screening methods: (1) Depth of each variation (DP)≥3, meaning that each allele was covered by at least three reads; (2) Variant/Reference Quality≥30, meaning that the alternate allele was supported by a minimum Phred mass fraction of 30; and (3) Genotype Quality (GQ)≥30, meaning that the genotype quality required a Phred quality score greater than 30. The mass score was given as the Phred-scale value, where Q = -10log_10_P (P is the probability of the base call being correct). We initially detected 976,733 potential SNPs and 36,846 potential InDels, leaving 565,979 SNPs and 13,357 InDels after the above series of strict filtering steps (S8 Table). Although this stringent threshold significantly reduced the number of genetic variants, we believe that it was helpful to eliminate false positives and ensure the accuracy of the results.

### Validation of differential genes by quantitative real-time PCR

**Fig 8:**
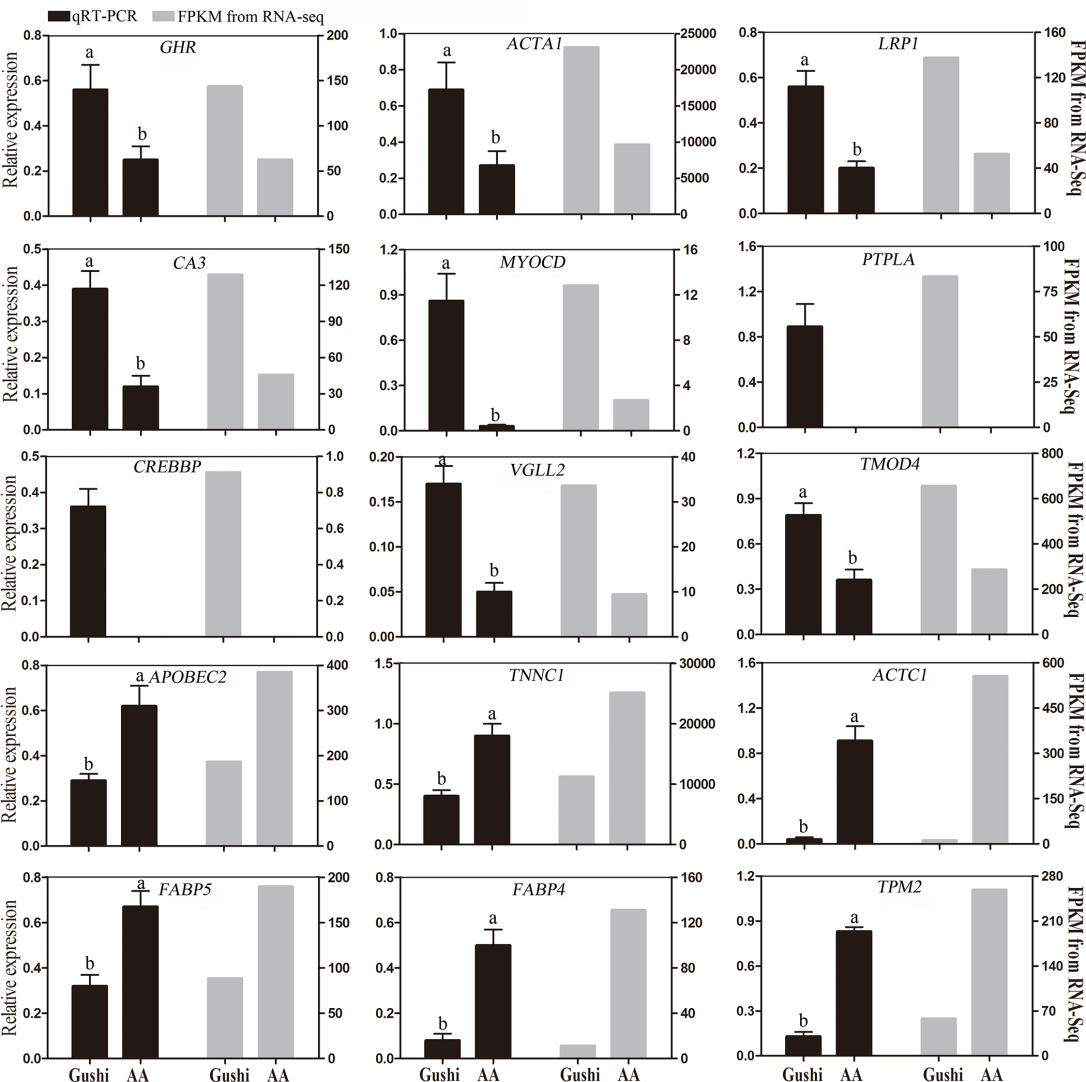
Validation of RNA-Seq data by qRT-PCR. Data were analyzed by the 2^–ΔΔCt^ method using GAPDH as a reference gene. The results are presented as fold changes in expression. Each column represents the means ± SEM from 3 biological replicates with each measurement repeated 3 times. Different lowercase letters indicate significant differences in expression levels between the two breeds (P ≤ 0.05). Black bars = qRT-PCR; Gray bars = FPKM from RNA-Seq.

To verify the accuracy of the RNA-Seq results for the transcriptome, 15 genes were randomly selected, including 9 significantly upregulated genes and 6 significantly downregulated genes. The expression levels of these 15 genes were quantified using qRT-PCR, with *GAPDH* as the internal reference gene. Each group had three biological repeats, and each sample was repeated three times. The expression level was calculated using the 2^-ΔΔCt^ method, and the values represent the means ± standard error (means ± SEM). The results showed that the vast majority of genes selected by qRT-PCR validation and sequencing data (Fig 8) had similar expression patterns, suggesting that the detection and expression abundance of genes in our transcriptome sequencing was highly accurate.

## Discussion

Construction of an RNA-Seq library is usually based on the oligo(dT)-based poly(A) enrichment method, which requires very high-quality and complete total RNA samples for sequencing. Moreover, this method has a large drawback in that it cannot capture poly(A)- transcripts or partially degraded mRNAs, thus leading to inadequate transcriptome results. Recent studies have shown that Ribo-Zero RNA-Seq can overcome these limitations by simultaneously capturing poly(A)- RNA and immature and partially degraded mRNAs from intact or partially degraded RNA. Ribo-Zero RNA-Seq and mRNA-Seq both provide efficient rRNA removal and uniform genomic coverage, while the former has unique advantages in terms of technical reproducibility[1, 40]. Thus, Ribo-Zero RNA-Seq is undoubtedly the best way to study transcriptomes, especially for organisms with completely annotated genomes, including the chicken.

Gushi chickens are a well-known local breed that is characterized by slow growth and high-quality meat, and AA broilers are representative of fast-growth broilers. Analyzing differences in the genes and regulatory pathways of these two breeds is important for elucidating the molecular mechanisms of AA broilers’ fast growth and Gushi chickens’ high meat quality. Increasing numbers of transcription analyses of different breeds are being performed [41–46].

We used the pool sequencing method to identify transcript groups in the breast tissue of Gushi chickens and AA broilers. Although pool sequencing cannot detect individual differences within the same breed, this method can cover more individuals; thus, the data obtained can provide more comprehensive information about the chest transcriptome of a certain breed, and pool sequencing can provide sufficient genetic material to facilitate analyses of SNPs, InDels, and alternative splicing[47, 48].

Transcriptome data can be used to analyze the genetic variation that occurs in the transcriptional region. A series of studies suggested that genetic variation could affect the phenotypic characteristics of an animal. In a previous study, 30,618 and 31,334 SNPs were detected by mRNA-Seq in different pooled samples using the criteria of at least 2 unique mapped reads supporting the polymorphic nucleotide and a quality score of ≥20[47]. To clarify the variability in transcription levels, large-scale SNP and InDel scans were performed in this study. We found that even if we adopted more stringent standards (mass fraction not less than 30 and minimum coverage not less than 3), the number of SNPs we obtained was still far greater than in previous reports. A large number of studies included large-scale SNP scanning analyses [49–55]. However, corresponding InDel analyses are still scarce. In this study, we detected 13,357 InDels. The large numbers of unannotated SNPs and InDels identified in this study will provide material for research into chicken genetic variation and thus contribute to chicken breeding. We found that the *CETP* intron region of Gushi chickens contained a two-base insertion. *CETP* was an upregulated gene associated with lipid metabolism and deposition. This insertion may be an important factor affecting the difference in meat quality between Gushi chickens and AA broilers.

A large number of DEGs were associated with fatty acid transport, metabolism and muscle development function, especially in the biological pathway analysis; transcripts of multiple groups of genes were significantly enriched in the FoxO, MAPK, and PPAR signaling pathways. IMF deposition involves a complex regulatory network controlled by polygenes. It has been reported that peroxisome proliferator-activated receptor gamma (*PPARG*)[56] and sterol regulatory element-binding transcription factor 1 (*SREBF1*, also termed *SREBP*) [57] are two important regulators of fatty acid metabolism. Through the screening of 45 key genes in fatty acid metabolism in dairy cows, Massimo Bionaz and Juan J Loor found that *PPARG*, *SREBF1* and *SREBF2* were at the core of a fatty acid metabolism control network and played an important role in the regulation of lipid metabolism[57].

The overexpression of either *PPARG* or *SREBP* increases the expression of genes related to fatty acid metabolism. The protein encoded by *PPARG* can promote the expression of Adipose differentiation-related protein (*ADRP*) and stearoyl-CoA desaturase (*SCD*) mRNA by binding to the promoter of the gene[56, 58]. The protein encoded by *SCD* is a key enzyme in the synthesis of monounsaturated fatty acids and conjugated linoleic acids in cells. There was a significant positive correlation between the expression of the *SCD* gene and the IMF content in different breeds and tissues. *SCD* is an important gene that affects liver fat metabolism[26]. In rodent liver, SREBF1 is a key transcriptional regulator in the cholesterol and FA synthetic pathways[59] that binds directly to target gene promoters including those of fatty acid synthase (*FASN*), acetyl-CoA carboxylase alpha (*ACC*) and *SCD* to regulate transcription[60]. However, the upregulation of *SCD* was induced much more strongly by the overexpression of *PPARG* than by *SREBF1*[56].

In Xinong Saanen goat mammary epithelial cells, *SREBP1* can promote the expression of genes related to fatty acid synthesis, including *FASN* and *ACC*, indicating that *SREBP1* plays a role in the regulation of fatty acid metabolism in the goat mammary gland. However, the expression level of *SCD* was not changed[61]. Similarly, in our results, the *SREBF1* gene was significantly upregulated, but there was no significant difference in the expression of the *SCD* gene. We hypothesized that the absence of significant changes in *SCD* expression may be associated with the significant downregulation of the *PPARG* gene.

Fatty acid binding protein 3 (*FABP3*), also known as heart-fatty acid binding protein (*H-FABP3*), is at the heart of the fatty acid metabolic regulation network and plays an important role in the regulation of fatty acid metabolism and IMF deposition. It was found that the expression level of *FABP3* was negatively correlated with the IMF content in the thigh[62]. Related research[63] showed that the expression of *FABP3* mRNA in the chest muscle did not differ between the Hetian-black chicken (HTBC) with excellent meat taste and the fast-growing three-yellow chicken (TYC). Similarly, there was no significant difference in the expression of *FABP3* in the two chicken breeds in our study. Differences in research results may be caused by differences in breeds and breeding environments. The *LPL* gene, which encodes a key enzyme in lipid metabolism, is mainly expressed in fat and skeletal muscle. Its expression level was positively correlated with IMF content in breast muscle[64]. In our study, although the PPAR pathway was not significantly enriched, the *LPL*, *FABP5* and solute carrier family 27 member 6 (*SLC27A6*) genes in this pathway were significantly reduced, which may have been caused by a significant reduction in *PPARG*. In short, the interaction between *PPARG* and *SREBP* at the core of the fatty acid metabolism regulation network is unclear and requires further study. The *CETP* gene plays an important regulatory role in promoting the synthesis or deposition of lipids in broilers (Tall, 1993; Bezaire, et al., 2005). In this study, expression level of the *CETP* gene was higher in the breast tissue of Gushi chickens than in that of AA broilers. The *CETP* gene may be involved in the molecular regulation of Gushi chicken quality formation.

The *CSRP3* gene is most highly expressed in skeletal muscle and cardiac muscle and plays an essential role in the proliferation and differentiation of striated muscle cells[65, 66] [67]. *MYL10*, *MYH7B*, *MYL3*, and *FGF3* were found to be DEGs associated with muscle development in Beijing-you chickens and AA broilers [33]. It was assumed that these DEGs contributed to the process of IMF deposition [33]. Most of these genes were downregulated in Gushi chickens compared with AA broilers and thus may contribute to the fast growth of AA broilers. All the DEGs discussed above may be candidate genes for meat quality and growth traits of Gushi chickens and AA broilers.

In conclusion, RNA-Seq was used to study the transcriptome data of breast tissue from Gushi chickens and AA broilers, and the genetic variation and variable splicing of gene sequences were analyzed comprehensively. Compared with AA broilers, more annotated genes and novel genes were detected in the breast tissue of Gushi chickens, revealing that Gushi chickens contain unique genetic information and indicating that the Gushi chicken genome warrants further study. The finding of more variable shear events and genetic information variation indicated that the breeding level of Gushi chickens is also lower than that of AA broilers. The DEGs included a large number of lipid metabolism- and muscle development-related genes, which may be involved in the molecular regulation of meat quality formation. The genes of AA broilers had higher expression levels than those of the Gushi chickens, which showed that AA broilers have the characteristics of fast growth and strong metabolism.

## Conclusion

This study used Ribo-Zero RNA-Seq technology to analyze the pectoral muscle transcriptome in different breeds of chicken. The results revealed DEGs, novel genes and pathway enrichment. These findings will be a valuable resource for biological investigations of muscle growth- and meat quality-related genes in chickens.

## Supporting information

S1 Table Specific primers used for qRT-PCR validation. (DOCX)

S2 Table The 10 highest expression genes in muscles of AA chickens and Gushi chickens. (XLSX)

S3 Table The different expression genes in muscles of AA chickens and Gushi chickens. (XLSX)

S4 Table The top 20 upregulated and downregulated genes in muscles of Gushi chickens compared with AA chickens. (XLSX)

S5 Table The GO enrichment of different expressed genes of Gushi chickens and AA chickens. (XLSX)

S6 Table The KEGG enrichment of different expressed genes of Gushi chickens and AA chickens. (XLSX)

S7 Table The novel genes in muscles of AA chickens and Gushi chickens. (XLSX)

S8 Table Indels in muscles of AA chickens and Gushi chickens. (XLSX)
