# Supplementary material for: Transcriptome analysis of the pectoral muscles of local chickens and commercial broilers using Ribo-Zero ribonucleic acid sequencing
Source: PLoS One. 2017 Sep 1;12(9):e0184115. doi: 10.1371/journal.pone.0184115 (PMC5581173; doi:10.1371/journal.pone.0184115)
Supplement: S1 Table — (DOCX) [file pone.0184115.s001.docx]

**S1 Table. Specific primers used for qRT-PCR validation**

| Gene | Primers (5'-3') | sizes (bp) |
| --- | --- | --- |
| *GHR* | F1: GACACTTACTTCACCACAGA | 200 |
|  | R1: CTCACATAGCCACAAGACA |  |
| *ACTA1* | F2: CTGTGCTATGTGGCTCTG | 156 |
|  | R2: AATGAAGGAAGGCTGGAAG |  |
| *LRP1* | F3: CACCAAGTGGAGCAGATG | 191 |
|  | R3: CCGTAGTCCGTGAAGAAC |  |
| *CA3* | F4: CTACGACAAGGAGAATGGA | 171 |
|  | R4: CCGTTGTTCAGGATGGTT |  |
| *MYOCD* | F5: CACTGATGGAACGGCTAC | 128 |
|  | R5: AGACTGCGACTGGTAACT |  |
| *PTPLA* | F6: GTCCAAGTGAGTTCAAGAATC | 104 |
|  | R6: ACAGTCCATACGACCAGAA |  |
| *CREBBP* | F7: CAGCCTCAGACACAGTTG | 177 |
|  | R7: GGATGTTGGAAGAGCAGTT |  |
| *VGLL2* | F8: ACTCGCCTTGTACTCCAA | 109 |
|  | R: GCACTCCTCCTCCTTGAT |  |
| *TMOD4* | F: CGTCAACCTCAACAACATC | 156 |
|  | R: TTCTCTGCCAGCATCTCA |  |
| *APOBEC2* | F: CACAGAGACACAACAGACT | 180 |
|  | R: GGTGAAGAAGAACAACAACA |  |
| *ACTC1* | F: ACTACCTACAACAGCATCAT | 200 |
|  | R: CCACCAATCCAGACAGAG |  |
| *TNNC1* | F: AAGAATGCTGATGGCTACA | 143 |
|  | R: AACTCGTCATAGTCAATCCT |  |
| *FABP5* | F: GGATGGCAAGAAGACTGTA | 157 |
|  | R: CCACTGGACTGAGTTGTT |  |
| *FABP4* | F: AGACCACAGCAGATGACA | 136 |
|  | R: CATTCCACCAGCAGGTTC |  |
| *TPM2* | F: CTTAGAAGACGAAGTGTATGC | 194 |
|  | R: AGAGTGAACAGGAGGAGTG |  |
